# Did You Enjoy It? The Role of Intensity-Trait Preference/Tolerance in Basic Psychological Needs and Exercise Enjoyment

**DOI:** 10.3389/fpsyg.2021.682480

**Published:** 2021-06-10

**Authors:** Diogo S. Teixeira, Filipe Rodrigues, Sérgio Machado, Luis Cid, Diogo Monteiro

**Affiliations:** ^1^Universidade Lusófona, Faculty of Physical Education and Sport, Lisbon, Portugal; ^2^Research Center in Sport, Physical Education, and Exercise and Health (CIDEFES), Lisbon, Portugal; ^3^Sport Science School of Rio Maior (ESDRM-IPSantarem), Rio Maior, Portugal; ^4^Life Quality Research Centre (CIEQV), Santarém, Portugal; ^5^Department of Sports Methods and Techniques, Federal University of Santa Maria, Santa Maria, Brazil; ^6^Research Center in Sport, Health and Human Development (CIDESD), Vila Real, Portugal; ^7^ESECS – Polytechnique of Leiria, Leiria, Portugal

**Keywords:** preference, tolerance, psychological needs, enjoyment, exercise

## Abstract

Promoting exercise regimens that aim at enhancing the quality of individuals’ subjective exercise experience can be challenging. Given the recent theoretical contributions regarding the possible interaction of exercise intensity-traits and several motivational variables, as well as their potential value for exercise adherence, the objective of this study was to examine the mediation role of basic psychological needs in the relationship between preference for and tolerance of exercise intensity and enjoyment. This cross-sectional study comprised a total of 160 exercisers (*Mage* = 34.12, *SD* = 9.23, 73 males) enrolled in several health clubs. All analyses were performed using SPSS v. 23.0/PROCESS v. 3.4. The results indicate that intensity-traits presented positive associations with enjoyment, and negative associations with all of needs frustration variables. A mediation role of needs frustration emerged in the intensity-traits and enjoyment associations that was analyzed according to relatable theoretical considerations.

## Introduction

In the last decades, significant evidence has indicated that regular exercise is capable of improving one’s physical and mental health (Pedersen and Saltin, 2015; Warburton and Bredin, 2017). Most common contexts of exercise practice worldwide are gymnasiums and health clubs (European Commission, 2018; International Health, Racquet and Sportsclub Association, 2020), and usually offer a wide variety of activities and possibilities for exercisers. In this context, exercise professionals conduct their work in accordance with the activity they are supervising, the exerciser’s goals, and general club management guidelines.

In these multifactorial exercising interactions, promoting the dynamics that aim at enhancing the quality of the individual’s subjective experience can be challenging. For that matter, enjoyment was highlighted as a significant determinant of intention to continue and exercise adherence (Nielsen et al., 2014; Calder et al., 2020; Chen et al., 2020). However, due to the intrinsic characteristics of the activities offered in health clubs, promoting exercisers enjoyment may not be easy.

Several approaches have been presented in the last two decades of research aiming to help professionals achieve this outcome. However, gym dropout rates are still alarmingly high, rounding 75% and 50% in the three and six months of initial practice, respectively (Edmunds et al., 2007; Buckworth et al., 2013; Sperandei et al., 2016; Radel et al., 2017). Considering the worrisome levels of sedentary behavior and physical inactivity worldwide (European Commission, 2018; Physical Activity Guidelines Advisory Committee, 2018; World Health Organization, 2018), health clubs may have an important role in the fight against this scourge, and contemporary multi-theoretical approaches may be helpful to address and promote exercise sustainability (Teixeira et al., 2020).

### The Role (and Promotion) of Enjoyment in Exercise

The impact of a behavior such as physical activity is emotionally and subjectively interpreted by individuals, gaining a positive and reinforcing meaning (i.e., when the activity is perceived as interesting, pleasant, and enjoyable), or a negative and avoidant value (i.e., if an activity is perceived as unpleasant, boring or uninteresting). Consequently, the perception of a certain activity appears to have a substantial effect on exercise engagement and commitment in the future (Jekauc and Brand, 2017; Rodrigues et al., 2021). As reported in literature, enjoyment can be understood as an experience that reflects generalized feelings of pleasure, liking, and satisfaction, and may reflect an intrinsically motivational factor for participation in physical activity (Moore et al., 2009; Nielsen et al., 2014). In several physical activity related contexts, enjoyment has presented positive associations with intention and adherence, as is the case for sports (Nielsen et al., 2014; Granero-Gallegos et al., 2017; Teixeira et al., 2019), physical education (Barr-Anderson et al., 2008; Gardner et al., 2017; Fin et al., 2019), exercise (Chen et al., 2020; Klos et al., 2020; Rodrigues et al., 2020), and leisure-time physical activity (Gardner et al., 2017).

A contemporary theoretical approach that has shown evidence of how motivational factors can support activity enjoyment is the Self-Determination Theory (SDT; Deci and Ryan, 1985; Ryan and Deci, 2017). This theory postulates that the regulation of a given behavior (e.g., physical exercise) can be performed across a motivational continuum ranging from non-self-determined or controlled regulations (i.e., amotivation, external and introjected regulations), to self-determined or autonomous regulations (i.e., identified and integrated regulations; intrinsic motivation). More importantly, SDT sustains the concept that the degree of internalization and integration of the behavior is influenced by how three basic and innate psychological needs (BPN) are satisfied or frustrated (Ryan and Deci, 2017). According to SDT, distinct elements can fulfill or thwart autonomy (i.e., one’s ability to choose their behavior and be in control of the activity), competence (i.e., one’s ability to succeed at challenging tasks and attain desired outcomes), and relatedness (i.e., one’s ability to establish meaningful interactions with others in a trustworthy and respectful manner), promoting differentiated outcomes. Generally, BPN satisfaction is related to well-being and personal growth, whereas BPN frustration is associated with ill-being and rumination (Ryan and Deci, 2017; Ntoumanis et al., 2020; Vansteenkiste et al., 2020).

For example, in exercise settings BPN satisfaction has been positively associated to several well-being indicators (e.g., enjoyment, positive affect) (Puente and Anshel, 2010; Teixeira and Palmeira, 2015; Teixeira et al., 2018a) and intention to continue exercising (Rodrigues et al., 2019b; Rodrigues et al., 2020). As for BPN frustration, Teixeira et al. (2018b) have shown a negative association with several well-being indicators (e.g., psychological well-being, positive affect). Additionally, this study supported previous claims made in the sports context (e.g., Bartholomew et al., 2011; Warburton et al., 2020), showing that need satisfaction and frustration can co-occur in several sport related contexts, justifying the importance to better understand contextual characteristics that may facilitate or hinder needs fulfillment.

Yet, with regard to enjoyment promotion, in a recent systematic review that aimed to analyze interventions that promote positive affect (and enjoyment) and physical activity, the authors have claimed that *“The most commonly used theory in this review is the SDT, and most studies incorporating it improve both PA [physical activity] and enjoyment”* (Klos et al., 2020, p. 11). However, the authors also suggest that, despite SDT being the most established theory focusing on affect and physical activity promotion, *“(*…*) more affect-oriented theories should be integrated into interventions to test and develop new approaches”* (Klos et al., 2020, p. 13).

In fact, the research conducted in the recent years has brought a new light to the hedonic approach in exercise. This theoretical development supports the idea that humans tend to sustain a behavior that they perceive and feel as pleasurable and enjoyable, and tend to avoid the activities that are painful or may cause discomfort (Ekkekakis and Zenko, 2016; Murphy and Eaves, 2016). This assumption leads to an idea that pleasurable experiences can support and promote sustainable exercise practice, with some studies and research providing evidence for these claims (Williams et al., 2008; Ekkekakis and Dafermos, 2012; Rhodes and Kates, 2015).

One particularly relevant factor related to a pleasurable experience obtained from exercise is intensity (Ekkekakis et al., 2011). Several studies have reported that increases in exercise intensity are related to the participants’ affective states, with higher intensities being generally associated with reduced pleasure or increased displeasure (Rose and Parfitt, 2007; Williams et al., 2008; Evmenenko and Teixeira, 2020; Rhodes and Kates, 2015).

However, this factor (i.e., intensity-pleasure relation) varies considerably among individuals. For that matter, some studies have measured individual differences in the preference for and tolerance of exercise intensity. They have shown that preference (i.e., a predisposition to select a particular level of intensity) and tolerance (i.e., a trait that influences one’s ability to continue exercising at a defined level of intensity) are linked to individual’s affective response to exercise and fitness performance (Ekkekakis et al., 2005, 2006, 2007; Hall et al., 2014; Jones et al., 2018). Some of these studies have shown that preference and tolerance were positively associated with total leisure-time exercise (Ekkekakis et al., 2008), exercise frequency (Ekkekakis et al., 2007; Teixeira et al., 2021), and could predict affective responses in high intensity exercise protocols (Jones et al., 2018; Box and Petruzzello, 2020).

However, and despite some studies providing evidence in favor of an intensity-guided exercise prescription aiming to promote distinct and beneficial behavioral, cognitive and emotional outcomes, a large gap still exists in other contexts, populations, and variables of interest. Some researchers suggest that preference and tolerance are positively related with enjoyment intra- and post-exercise, which seems to be in line with the hedonic approach, supporting the idea that exercise prescription should be adjusted to individual’s intensity preferences in order to improve continuous exercise adherence (Schneider and Graham, 2009; Box and Petruzzello, 2020). However, some authors recall that the understanding of how these intensity-trait differences influence in-task exercise affect and enjoyment is still limited, and more research is warranted to better understand these relations and how to apply them in real-life settings (Ekkekakis et al., 2008; Box and Petruzzello, 2020; Teixeira et al., 2021).

With regard to the context of practice, and to the best of our knowledge, only one study has addressed the role of intensity-traits in health club exercisers. The study showed that preference and tolerance were, in general, positively associated with exercise frequency, habit, vitality and other well-being variables, particularly when exercisers reported that their training intensity was in agreement with the one they prefer and can tolerate (Teixeira et al., 2021). These results tend to suggest that elaborating an exercise prescription that focuses on an intensity regulation aligned with the exerciser’s characteristics and preferences, may present differentiated and positive outcomes, as suggested by some previous research (e.g., Dishman et al., 1994).

### Present Study

Given the aforementioned evidence and framework, some theoretical considerations need a better understanding and exploration in future scientific endeavors. The role of enjoyment as a predictor of exercise engagement and intentions to continue practice seems to be well-established in the literature, and particularly in the exercise-related context. However, given the myriad of contextual characteristics in health club settings, the promotion by professionals of individual enjoyment in a particular exercise regimen may be challenging.

The exploration of how a contextual intervention focused on the two abovementioned intensity-traits can sustain and improve enjoyment seems plausible when considering the existing evidence. This seems particularly relevant taking into account that millions of exercisers are enrolled in health club activities (International Health, Racquet and Sportsclub Association, 2020) and that these exercise contexts still suffer high dropout rates in the first 6 months of practice.

From another point of view, the association between BPN (both direct and indirect) and enjoyment seems rather clear in most contexts, and strong evidence supports interventions based on the SDT framework. However, as pointed out by Klos et al. (2020), more affective-guided theories should be integrated and tested in new research approaches and interventions (e.g., SDT-based) that aim to improve participation in physical activity.

To the best of our knowledge, no study has ever focused on the possible interconnection of the intensity-traits of preference/tolerance and BPN satisfaction/frustration. It is hypothesized that the intensity-traits agreement with the exercise training should be supportive of needs satisfaction. Grounded in SDT, and particularly in health club settings, professionals’ interpersonal behaviors and respective interactions with exercisers can promote or thwart BPN (Ryan and Deci, 2017; Rodrigues et al., 2018). Thus, it can be expected that an exercise prescription that is adjusted to an individual’s pre-determined intensity preference and tolerance might be a promoter of all of the BPN, a hypothesis that should receive attention in future research. Particularizing, the operational definition of an adjusted intensity may be obtained through a guided self-selected intensity or through the definition of an adequate intensity range based on transitional affective changes (e.g., through the circumplex model of affect), thus promoting the ability to choose one’s behavior and be in control of the activity, consequently being perceived as a facilitator of autonomy need satisfaction. Additionally, activities performed at a self-selected intensity may aid developing competence and mastery that is adjusted to the actual level of ability, capabilities and interest (i.e., to promote the ability to succeed at challenging tasks and attain desired outcomes), thus promoting individual perception of competence satisfaction. Finally, if exercise prescription and supervision is tailored in accordance with the intensity-traits characteristics expressed by the exerciser to the professional, it may further be reflected in the form of individual concern, relevance, and emotional support by the professional. In practice, it may imply the ability to establish meaningful interactions with others in a trustworthy and respectful manner, reinforcing the perception of relatedness need satisfaction. Moreover, considering the co-occurrence of BPN satisfaction and frustration in health clubs (Teixeira et al., 2018b), it can be hypothesized that a deliberate or purposeful disregard for these characteristics may be reflected in the exerciser’s perception of BPN frustration (an active process of a controlling behavior; e.g., the “no pain, no gain” dyad).

Additionally, in a recent work developed by Vansteenkiste et al. (2020) regarding the role of the BPN theory and future research advances, some focus was given to understanding what can be considered as need-relevant conditions. It is posited that, to some degree, BPN are influenced by other psychological characteristics and personality traits, and that some endeavors should be done to further understand these possible interactions. As stated previously, both preference and tolerance represent personality traits related to exercise intensity. Although, to our knowledge, no study has tested this concept, it is hypothesized that an intensity-trait and needs relation should exist, with this being particularly relevant to future assessment of intensity-guided exercise prescriptions.

All in all, these theoretical assumptions seem to be in line with a systematic review aimed to theoretically explore the maintenance of behavior change (Kwasnicka et al., 2016). In the study conclusions, it is suggested that in order to sustain health-related behaviors, more focus should be given to promoting behavioral options that are enjoyable and facilitating individual behavior self-regulation, as is the case of self-monitoring behavior (e.g., teaching of exercise intensity self-regulation).

Thus, the aim of this exploratory cross-sectional study is to analyze the relationship between preference for and tolerance of exercise intensity and BPN and enjoyment, and to test a possible mediation role of the psychological needs in the intensity-trait and enjoyment exercise dynamics. It is expected that intensity-traits would present positive associations with enjoyment that should be mediated by the BPN satisfaction and frustration. Given the underexplored theoretical and contextual dynamics of the proposed variables, this study could add some support to the existing literature for future longitudinal and experimental research aiming to improve exercise sustainability through the prism of hedonic theory and SDT.

## Method

### Participants and Procedures

A total of 160 exercisers (*M*_*age*_ = 34.12; *SD* = 9.23; 73 males) with a mean of 10.14 years of experience (*SD* = 5.6) enrolled in several health clubs (*n* = 8) participated voluntarily in this study. The clubs were randomly selected to be invited to participate in the study. The participants were engaged in individual (59%) (e.g., strength training, personal training), group (29%) (e.g., dance, choreographed aerobics), and mixed training sessions (12%). For participation, individuals had to be ≥ 18 years, be enrolled in health clubs, and had had at least 60 minutes of weekly practice during the previous three months.

When the research plan was approved, health club managers were contacted and provided with study information and authorization requests. After obtaining permission, the recruitment of potential participants was conducted through advertisements at the health clubs’ reception desks, through fitness professionals’ contacts, and via health clubs mailing lists. The questionnaires could be completed in two forms: in person or via Google forms accessed with a QR-code. At the beginning of each questionnaire, a study explanation and expected participation was provided, clarifying that the participant could cease to participate at any moment without any repercussion, and that the confidentiality of the information would be ensured. The contact from the corresponding researcher was also provided in order to clarify any additional questions that could emerge regarding the study objective. Written consent was obtained from each exerciser individually in order to access the questionnaires and enroll in the present study. This sample is part of a larger ongoing study approved by the ethics committee of the Faculty of Physical Education and Sport of Lusófona University. All research-related procedures were developed in accordance with Helsinki declaration and later amendments.

### Measures

Preference for and Tolerance of Exercise Intensity (PRETIE-Q-PT; Teixeira et al., 2021). The PRETIE-Q-PT was used to measure preference (e.g., “*I would rather work out at low intensity levels for a long duration rather than at high-intensity levels for a short duration*”) and tolerance (e.g., “*Feeling tired during exercise is my signal to slow down or stop*”) traits related to exercise intensity. The Portuguese version contains 10 items (5 for each construct), answered on a 5-point bipolar Likert scale anchored from 1 (I totally disagree) and 5 (I totally agree). This questionnaire was validated in a health club exercisers sample and presented good psychometric properties. In this study, both subscales presented adjusted reliability scores (Cronbach’s alpha; preference α = 0.86; tolerance α = 0.71).

The Basic Psychological Needs Satisfaction and Frustration Scale in Exercise (BPNSFS-E; Rodrigues et al., 2019a). Grounded in SDT, this scale contains 24 items that measure BPN satisfaction (12 items: e.g., autonomy: *“When I exercise I feel a sense of choice and freedom in the exercises I undertake”*; competence: *“When I exercise I feel confident that I can do exercises well”*; relatedness: *“When I exercise I feel connected with others in the gym”*), and BPN frustration (12 items: e.g., *“When I exercise I feel forced to do training sessions I would not choose to do”*; competence: *“When I exercise I feel disappointed with my performance”*; relatedness: *“When I exercise I feel that the relationships I have at the gym are just superficial”*) in the exercise context. Answers are given using a 5-point bipolar Likert scale ranging from 1 (Totally disagree) to 5 (Totally agree). For study purposes, analysis were made with the individual needs for descriptive and correlational tests. For mediation analysis two composite factors were created. One composite factor for needs satisfaction (BPNS) and one composite factor for needs frustration (BPNF) were calculated by averaging the three needs responses. This instrument and procedure has been used in several studies in the exercise context according to research purposes, and has been used particularly in analyses that are dependent of lower samples sizes (e.g., Markland and Tobin, 2010; Teixeira et al., 2018b). In present study sample, all subscales presented good reliability (Cronbach’s alpha ranged between 0.74 and 0.88; Table 1).

**TABLE 1:** Descriptive statistics and correlation analysis of exercise intensity-traits, basic psychological needs, and enjoyment.

	M	SD	α	1	2	3	4	5	6	7	8	9	10	11
(1) Preference	17,86	4,72	0.86	−										
(2) Tolerance	16,10	4,12	0.70	0.624***	−									
(3) Autonomy satisfaction	16,93	2,87	0.82	0.087	0.048	−								
(4) Competence satisfaction	17,39	2,38	0.86	0.147	0.164*	0.748***	−							
(5) Relatedness satisfaction	16,44	3,35	0.79	–0.048	–0.090	0.560***	0.586***	−						
(6) Autonomy frustration	6,84	2,96	0.88	−0.256***	−0.207**	−0.571***	−0.458***	−0.161*	−					
(7) Competence frustration	7,19	3,17	0.74	−0.223**	−0.190*	−0.474***	−0.439***	−0.214**	0.631***	−				
(8) Relatedness frustration	6,36	2,71	0.78	–0.137	0.022	−0.317***	−0.377***	−0.488***	0.412***	0.401***	−			
(9) Satisfaction global	16,93	2,49	0.82	0.057	0.031	0.878***	0.874***	0.853***	−0.441***	−0.425***	−0.462***	−		
(10) Frustration global	6,77	2,39	0.74	−0.259***	−0.162*	−0.561***	−0.519***	−0.342***	0.846***	0.852***	0.727***	−0.539***	−	
(11) Enjoyment	46,48	8,26	0.91	0.287***	0.197*	0.622***	0.564***	0.449***	−0.589***	−0.437***	−0.437***	0.622***	−0.599***	−

* *p* < 0.05; ** *p* < 0.01; *** *p* < 0.001; α = Cronbach’s alpha.

The Physical Activity Enjoyment Scale Portuguese version (PACES; Teques et al., 2017). This 8-item scale (e.g., *“It is fun”*) evaluates the level of agreement of exercise enjoyment. The stem asked participants to answer eight questions regarding *“How do you feel at the moment about you are doing exercise”*. It is answered using a 7-point bipolar scale ranging from 1 (Totally disagree) to 7 (Totally agree). Previous studies support the use of this scale to assess enjoyment in this context and with Portuguese exercisers (Rodrigues et al., 2020). In present sample, the reliability score (Cronbach’s alpha) is considered adequate; enjoyment Cronbach’s alpha α = 0.91).

### Statistical Analysis

Data were screened for analysis assumptions, descriptive statistics and bivariate correlations. Participants with more than 5% of absent data were removed from further analysis. In questionnaires with less than 5% of missing data (*n* = 3; 1.875%), multiple imputation procedures were applied (Allison, 2000). The level of statistical significance was set at *p* < 0.05. These calculations were performed using IBM SPSS Statistics v. 23.0 for Mac (IBM Co., United States).

For mediation analysis purposes, the SPSS PROCESS software V. 3.4 was used. Following Hayes (2018) recommendations, a multiple mediator analysis model (model 4) was used to test the variables interaction and possible mediation effects. This procedure estimates the direct effect of the independent variable (intensity-trait) on the dependent variable (enjoyment), and the indirect effect through multiple mediators (two: BPNS and BPNF). Bootstrap with 5,000 samples was used and the confidence interval (95%) estimate was calculated. Significant indirect effects were considered if 95% CI did not include zero (Hayes, 2018).

In mediation models with two or more independent variables, highly correlated scores may have suppressing effects. Given previous studies, particularly in the validation of preference and tolerance traits, a moderate to high correlation often emerge (e.g., Ekkekakis et al., 2005; Hall et al., 2014). Thus, and according to Hayes (2018), the selection of several independent variables in one model or the use of several models with only one independent variable while controlling for possible interactions (i.e., covariates), should be decided after testing both possibilities and considering research purposes and theoretical assumptions, being a particularly relevant decision when the independent variables are highly correlated. Given the current study *r* = 0.62, *p* < 0.05 (Table 1), and after testing both possibilities, independent models were developed for further analyses. Considering that in both situations the results presented the same trend in results and directions, the decision was made to present the models with only one independent variable, allowing for a better understanding of each variable interaction in the proposed models.

## Results

Preliminary analysis revealed no violation of normal distribution. In Table 1, descriptive statistics and correlational analyses of the studied variables are presented.

As seen, preference and tolerance presented some significant associations. Firstly, preference and tolerance were positively associated with enjoyment (*r* = 0.29, *p* < 0.001; *r* = 0.20, *p* < 0.05, respectively). Secondly, preference did not present associations with needs satisfaction (autonomy *r* = 0.09, *p* = 0.28; competence *r* = 0.15, *p* = 0.06; relatedness *r* = −0.05, *p* = 0.55; BPNS global *r* = 0.06, p = 0.48), but presented a negative association with autonomy frustration (*r* = −0.26, *p* < 0.001), competence frustration (*r* = −0.22, *p* < 0.01) and BPNF global (*r* = −0.26, *p* < 0.001). No association was found for relatedness frustration (*r* = −0.14, *p* = 0.09). Thirdly, tolerance was positively associated with competence satisfaction (*r* = 0.16, *p* < 0.05) but not with autonomy satisfaction (*r* = 0.05, *p* = 0.55), relatedness satisfaction (*r* = −0.09, *p* = 0.26) or BPNS global (*r* = 0.03, *p* = 0.70). Regarding tolerance and BPN frustration, negative associations were detected with autonomy (*r* = −0.21, p < 0.01), competence (*r* = −0.19, *p* < 0.05) and BPNF global (*r* = −0.16, *p* < 0.05). No association was found for relatedness frustration (*r* = 0.02, *p* = 0.78).

Enjoyment also presented positive associations with each need satisfaction and global score of BPN satisfaction (*p* < 0.05) and negative associations with each need frustration and global score of BPN frustration (*p* < 0.05). Cronbach’s alphas were above acceptable (> 0.70), suggesting adequate internal consistency across study variables.

**FIGURE 1:**
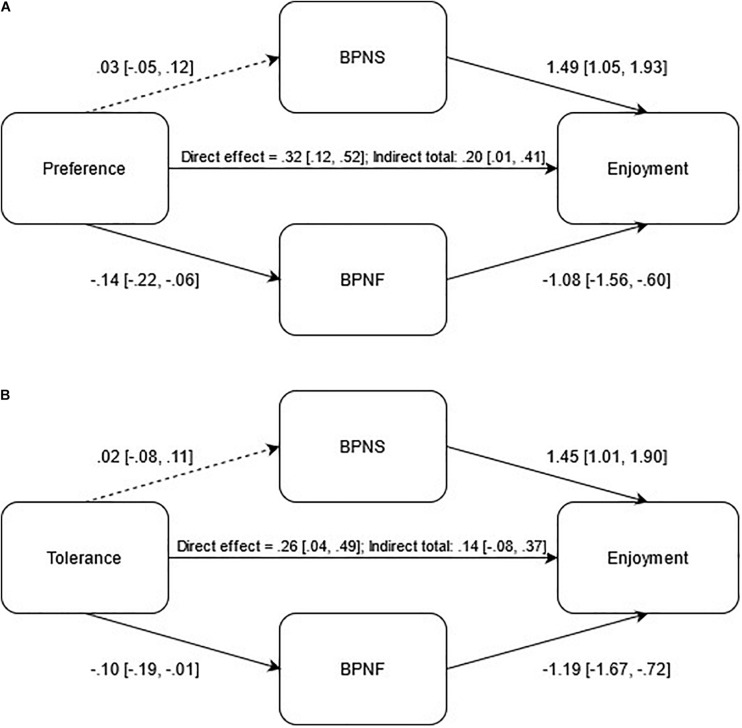
Direct and indirect effects analysis of basic psychological needs satisfaction and frustration in the relationship between preference **(A)** and tolerance **(B)** on exercise enjoyment. *Note*. BPNS – Basic Psychological Needs Satisfaction; BPNF – Basic Psychological Needs Frustration; Dashed lines – nonsignificant effect.

In Figure 1 are shown the tested mediation models. It is possible to observe that in model 1a there was a significant direct effect (preference-enjoyment; β = 0.32 [0.12, 0.52]) and, with the exception of the association between preference and BPN satisfaction (*p* = 0.44), all indirect paths presented significant values (all *p* < 0.05). The indirect total effect also presented a significant association (β = 0.20 [0.01, 0.41]). Regarding model 1b, there was also a significant direct effect (tolerance-enjoyment; β = 0.26 [0.04, 0.49]). All indirect paths followed the same pattern previously observed in model 1a (all *p* < 0.05; tolerance-BPN satisfaction *p* = 0.72), with the exception that the indirect total effect was not significant (β = 0.14 [−0.08, 0.37]).

## Discussion

The objective of this study was to examine the mediation role of basic psychological needs in the relationship between preference for and tolerance of exercise intensity and enjoyment. In general, both intensity-traits measured presented positive associations with enjoyment and negative associations with global experience of BPN frustration. Also, a significant total indirect effect was reported in the preference mediation model.

Previous studies have suggested that a hedonic approach to exercise may be a promoter of enjoyment (Ekkekakis and Zenko, 2016; Murphy and Eaves, 2016; Calder et al., 2020), and that the preference/tolerance intensity-traits may be related to this state (Ekkekakis et al., 2008; Box and Petruzzello, 2020). The present results seem to support the concept that intensity-traits are to some extent associated with enjoyment (small-to-moderate effect). Considering known associations between intensity and pleasure, it is expected that increases in exercise intensity would promote a better affective response until a specific point/moment, where inter-individual variability seems to differentiate the affective outcome dependent on exercise intensity (Ekkekakis et al., 2011; Ladwig et al., 2017; Evmenenko and Teixeira, 2020). Considering that enjoyment reflects positive feelings about exercise, it is plausible that a better affective response would be related to a better individual perception of a joyful activity or exercise experience (Raedeke, 2007; Rodrigues et al., 2021).

On an additional note, preference for and tolerance of higher exercise intensities may “protect” exercisers from moments or possible activities (purposefully or unintentionally) that expose them to unpleasant or disliked exercise intensities (e.g., higher tolerance), thus contributing to a better individual perception of exercise enjoyment. This would not be the case for low-to-moderate intensity preference and tolerance exercisers, which may be more prone to be exposed to unpleasant exercise intensities and, therefore, have a differentiated contribution to enjoyment. As seen in the study results, the preference (*M* = 17.86; *SD* = 4.72) and tolerance (*M* = 16.10; *SD* = 4.12) scores are slightly over the scale midpoint (5 - 25), reflecting an average preference/tolerance for moderate-intensity activities. This may suggest that this sample of exercisers, on average, does not have the same affective valence “flexibility” toward high-intensity ranges than a sample presenting higher preference/tolerance scores. This approach has been previously theorized (e.g., Raedeke, 2007) and has recently received some focus in a sample of 245 regular exercisers exposed to high-intensity body weight circuit (Box and Petruzzello, 2020). In this study, those with high intensity preference and tolerance traits reported exercise to be more pleasant and enjoyable than the lower-intensity preference counterparts during and after the high-intensity circuit.

Additionally, when comparing both groups in a light-intensity condition, high-preference/tolerance exercisers presented lower affective valence variability compared to the low intensity-preference group, further supporting a wider intensity range possibility for high preference and tolerance-trait exercisers compared to the low-intensity. This may justify different approaches when prescribing exercise intensities aiming to sustain enjoyment, given that different groups present distinct ranges of intensity-dependent pleasurable experiences (Box and Petruzzello, 2020).

Regarding the relationship between BPN and enjoyment, the results tend to support the existing evidence. The BPN satisfaction and BPN frustration presented positive and negative associations with enjoyment, respectively, which has previously been reported in these particular settings (e.g., Teixeira et al., 2018b; Rodrigues et al., 2020).

An additional aspect that is of particular relevance is the explored association between intensity-traits and BPN. As theoretically proposed, contextual characteristics and personality traits may be significantly related to BPN fulfillment (Ryan and Deci, 2017). The present results suggest that the intensity-traits have a negative association with BPNF (autonomy, competence, and BPNF global for both traits), and a positive association between tolerance and competence satisfaction. These outcomes are new to this field of research and possible meanings and explanations warrant further investigation. However, it is hypothesized that when the exercise intensity is aligned with individual preference and tolerance, this would be a positive predictor of BPN satisfaction. As posited by SDT, interpersonal and contextual characteristics can be interpreted as being supportive or controlling, thus influencing satisfaction or frustration of needs (Ryan and Deci, 2017). In this particular set, only the tolerance-competence satisfaction association was detected, that somehow aligns with the previous hypotheses. However, given the high scores of BPN satisfaction in the present sample, and the high participants exercise experience (*M*age = 10.14 years; *SD* = 5.6), which reflects a sustained and prolonged exercise practice, a *celling effect* may justify the absence of significant associations results. This may be further supported by the fact that the intensity-traits are negatively associated with needs frustration and that these partially mediate the trait-enjoyment relation (preference model). In regular exercisers (and in all individuals, with differentiated outcomes) it can be assumed that a deviation of the habitual exercise dynamics (e.g., exercise intensity) may have a deleterious effect on enjoyment, particularly if they perceive that that deviation is caused by an increase of controlling behaviors (Gunnell et al., 2013; Rodrigues et al., 2019c).

Another hypothesis that warrants further investigation is related to the fact that an exercise intensity-traits level of agreement with the current training intensity prescription may reflect distinct outcomes. For example, Teixeira et al. (2021) have shown that both intensity-traits present positive associations with cognitive, affective, and behavioral outcomes in a sample of 445 regular health club exercisers. However, when they analyzed the subgroups where the individual preference or tolerance where not in agreement with the exercise intensity prescribed, positive associations, in general, became non-significant. This may present the hypothesis that the level of disagreement between the actual training and the preferred/tolerated intensity, even in regular exercisers, may have a particularly deleterious effect on several outcomes. Thus, it can be suggested that the level of the intensity-traits and training agreement may act as a moderator in behavioral, affective and cognitive outcomes, possibly influencing motivational factors. These hypothesis, however, warrants further exploration for future contextual interpretation and applications.

Additionally, it was proposed that indirect effects would exist in the relationship between intensity-traits and enjoyment through BPN satisfaction and frustration. When analyzing the indirect paths in the models, both intensity-traits showed negative associations with BPNF, and BPNF presented negative associations with enjoyment. Both models indirect paths through BPNF and total indirect effect in the preference model were significant, thus suggesting relevant interactions between constructs. As such, both models suggest that the intensity-traits could be relevant to the promotion of a better exercise motivational quality, being aligned with previous recommendations for using multi-theoretical approaches aiming to improve exercise participation and sustainability (Ekkekakis and Zenko, 2016; Klos et al., 2020).

Considering the need-relevant conditions that may be dependent on personality and contextual factors (Vansteenkiste et al., 2020; Rodrigues et al., 2018), this intensity-traits preliminary exploration may add new insights on an improved BPN fulfillment in gyms and health clubs, further enriching the understanding of direct and indirect effects related to enjoyment.

### Limitations and Future Studies

Despite these interesting results, current evidence should be considered and interpreted based on existing limitations. Theoretically, variable interactions and models proposed in this study would benefit from a closer look grounded in dual-process theories. As proposed by several authors (Bluemke et al., 2010; Evans and Stanovich, 2013; Brand and Ekkekakis, 2018), the approached explained in the proposed theoretical models deal with automatic and reflective processes that can help to better understand the affective valuation of exercise (type-I processing) (e.g., core affect), or cognitive appraisal (e.g., of the stimulus; of the BPN perceptions). Particularly, regarding affective valuations, these can be the result of an emotion experienced and mediated by cognitive appraisals, or as a result of core affective reactions (e.g., bodily sensations), thus independent of cognitive evaluation. This exploration would also be aligned with the previously suggested multi-theoretical approaches addressed to promote the exercise sustainability.

Methodologically, the sample size and cross-sectional nature restricts the understanding and adequate expression of given results. Considering that this is an emerging line of research, future efforts should be made to replicate these findings with a larger sample, in different cultures, and with other robust statistical approaches.

Finally, contextually, a deeper understanding of the preference and tolerance relation with health, well-being, and performance indicators is needed for adequate contextual characterization, interpretation, and possible intervention guided to promote a pleasurable exercise experience. Additional focus could be given to the level of agreement between individual preference/tolerance and the training regimen, in order to assess the hypothesis of a detrimental effect of intensity-traits negligence.

## Conclusion

In the current exploratory study, the preference and tolerance intensity-traits presented positive associations with competence satisfaction (only tolerance) and enjoyment, and negative associations with needs frustration. In the mediation models, the indirect effects through BPNF, as for the total indirect effects (in preference model), were significant. Results tend to suggest a possible interaction between intensity-traits and enjoyment, which are partially mediated by BPNF.

## Data Availability Statement

The datasets presented can be made available on reasonable request by contacting the leading author (diogo.teixeira@ulusofona.pt).

## Ethics Statement

The studies involving human participants were reviewed and approved by Ethics committee of the Faculty of Physical education and Sport, ULHT. The patients/participants provided their written informed consent to participate in this study.

## Author Contributions

DT was responsible for study design and conceptualisation. DT and DM were involved in data collection. DT and FR wrote the manuscript. SM, LC, and DM reviewed and critiqued the manuscript. All authors made relevant contributions and approved the final version of the manuscript.

## Conflict of Interest

The authors declare that the research was conducted in the absence of any commercial or financial relationships that could be construed as a potential conflict of interest.
